# Hepatic glucose metabolic responses to digestible dietary carbohydrates in two isogenic lines of rainbow trout

**DOI:** 10.1242/bio.032896

**Published:** 2018-05-01

**Authors:** Xuerong Song, Lucie Marandel, Mathilde Dupont-Nivet, Edwige Quillet, Inge Geurden, Stephane Panserat

**Affiliations:** 1 INRA, Univ Pau & Pays de l'Adour, UMR1419 Nutrition Metabolism and Aquaculture, Aquapôle, F-64310 Saint-Pée-sur-Nivelle, France; 2 State Key Laboratory of Freshwater Ecology and Biotechnology, Institute of Hydrobiology, Chinese Academy of Sciences, Wuhan, Hubei 430072, China; 3 University of Chinese Academy of Sciences, Beijing 100049, China; 4 GABI, INRA, AgroParisTech, Université de Saclay, 78350 Jouy-en-Josas, France

**Keywords:** Fish nutrition, Carnivorous, Genetics, Metabolism pathway, Gene expression

## Abstract

Rainbow trout (*Oncorhynchus mykiss*) was recognized as a typical ‘glucose-intolerant’ fish and poor dietary carbohydrate user. Our first objective was to test the effect of dietary carbohydrates themselves (without modification of dietary protein intake) on hepatic glucose gene expression (taking into account the paralogs). The second aim was to research if two isogenic trout lines had different responses to carbohydrate intake, showing one with a better use dietary carbohydrates. Thus, we used two isogenic lines of rainbow trout (named A32h and AB1h) fed with either a high carbohydrate diet or a low carbohydrate diet for 12 weeks. We analysed the zootechnical parameters, the plasma metabolites, the hepatic glucose metabolism at the molecular level and the hormonal-nutrient sensing pathway. Globally, dietary carbohydrate intake was associated with hyperglycaemia and down regulation of the energy sensor Ampk, but also with atypical regulation of glycolysis and gluconeogenesis in the liver. Indeed, the first steps of glycolysis and gluconeogenesis catalysed by the glucokinase and the phospenolpyruvate carboxykinase are regulated at the molecular level by dietary carbohydrates as expected (i.e. induction of the glycolytic *gck* and repression of the gluconeogenic *pck*); by contrast, and surprisingly, for two other key glycolytic enzymes (phosphofructokinase enzyme – *pfkl* and pyruvate kinase – *pk*) some of the paralogs (*pfklb* and *pklr*) are inhibited by carbohydrates whereas some of the genes coding gluconeogenic enzymes (the glucose-6-phosphatase enzyme *g6pcb1b* and *g6pcb2a* gene and the fructose1-6 biphosphatase paralog *fbp1a*) are induced. On the other hand, some differences for the zootechnical parameters and metabolic genes were also found between the two isogenic lines, confirming the existence of genetic polymorphisms for nutritional regulation of intermediary metabolism in rainbow trout. In conclusion, our study determines some new and unexpected molecular regulations of the glucose metabolism in rainbow trout which may partly lead to the poor utilization of dietary carbohydrates and it underlines the existence of differences in molecular regulation of glucose metabolism between two isogenic lines which provides arguments for future selection of rainbow trout.

## Abstract (precis)

**Summary:** Using isogenic lines, this study determines some new, unexpected molecular regulation of the glucose metabolism in rainbow trout, which may partly lead to the poor utilization of dietary carbohydrates.

## INTRODUCTION

The rapid growth of aquaculture production over the last few decades has led to new challenges related to improving its sustainability. Among these, the improvement of fish nutrition and diet formulation are two major concerns (Naylor et al., 2009). Carbohydrates are regarded as an economical food source in fish farming, because their protein-sparing effect can diminish the level of dietary proteins in aquafeed (Shiau and Peng, 1993; Erfanullah and Jafri, 1995; Shiau and Lin, 2001). However, carnivorous fish, such as rainbow trout (*Oncorhynchus mykiss*), are considered to be poor users of carbohydrates, and tolerate no more than 20% of digestible carbohydrates in their diet (NRC, 2011). Indeed, the main consequence of this poor metabolic use is the persistent postprandial hyperglycaemia in fish fed with carbohydrates (Bergot, 1979; Enes et al., 2009; Skiba-Cassy et al., 2013). The low metabolic use of dietary carbohydrates has been proposed to originate from a poor molecular regulation of hepatic metabolic pathways by dietary carbohydrates including (1) a low efficiency of glycolysis (Sundby et al., 1991; Panserat et al., 2000a), (2) a lack of regulation of the endogenous glucose production through gluconeogenesis (Marandel et al., 2015; Panserat et al., 2000b and 2001), or a low induction of lipid synthesis (lipogenesis) from glucose (Panserat et al., 2009; Polakof et al., 2011 and 2012). However, more research into a better understanding of molecular mechanisms underlying the poor ability of carnivorous fish to use dietary carbohydrates is still needed.

Usually, in the aquafeed formulation dedicated to carnivorous fish, the increase in digestible carbohydrates content is balanced by a decrease in dietary protein levels (Kamalam et al., 2017). However, we know that dietary protein can strongly affect glucose metabolism in rainbow trout (Brauge et al., 1995; Seiliez et al., 2011) and that insulin-regulated gene expression is more responsive to dietary protein intake than carbohydrate intake (Seiliez et al., 2011; Dai et al., 2015). For this reason our objective is, for the first time, to analyse the molecular regulation of glucose metabolism by dietary carbohydrates alone (without variation of dietary protein level). Moreover, it is also important to analyse the nutritional regulation of the different trout paralogs because the salmonid-specific four whole-genome duplication event (Ss4R), occurring at the radiation of salmonids, is associated with the retention of a high number of duplicated genes in the trout genome (Berthelot et al., 2014). Indeed, these retained duplicated genes can evolve and lead to sub- or neo-functionalisations. One obvious example is the case of the five glucose-6-phosphatase paralogous genes which were found to be differently regulated in trout by dietary carbohydrates *in vivo* (Marandel et al., 2015) and insulin alone or combined with a high level of amino acid and/or glucose *in vitro* in hepatocytes (Marandel et al., 2016), proving that an individual analysis of the different gene paralogs in glucose metabolism (glycolysis, gluconeogenesis, lipogenesis, glucose transport) is needed.

Various different strategies have been explored to increase the level of carbohydrates in rainbow trout diet, which mostly focused on variations of nutritional factors (botanical origin of starch, dietary levels of carbohydrates, interactions between nutrients) or environmental factors (temperature, salinity, photoperiod) (Hung and Storebakken, 1994; Yamamoto et al., 2001; Kamalam et al., 2017). Genetic selection could be one of the new strategies to overcome the metabolic (nutritional) bottlenecks of carbohydrate utilization. Indeed, Mazur et al. (1992) revealed that different strains of chinook salmon (*Oncorhynchus tshawytscha*) showed significant differences in glucose utilization. Recently, the study of two rainbow trout lines divergently selected for their muscle lipid content (Quillet et al., 2005) showed that the fat line had a higher capability to use glucose than the lean line, linked to the enhancement of hepatic glycolysis, glycogen storage and lipogenesis (Kolditz et al., 2008; Skiba-Cassy et al., 2009; Kamalam et al., 2012; Jin et al., 2014). Therefore, searching for the potential genetic variability in glucose metabolism and its use in rainbow trout is an important step for future improvement in the use of dietary carbohydrates by carnivorous fish. Isogenic trout lines are powerful tools to study environmental genetic interaction (due to the high level of intra-line homogeneity) as reflected in previous studies (Quillet et al., 2007; Dupont-Nivet et al., 2012; Geurden et al., 2013; Sadoul et al., 2017). In the present experiment, two heterozygous isogenic lines of rainbow trout, defined as A32h and AB1h, were obtained by mating homozygous females from the same isogenic line B57 (thus all these females are genetically identical) with individual homozygous males of both lines A32 and AB1. In fact, AB1 had already been tested for plant-protein source utilization (Dupont-Nivet et al., 2009) and, surprisingly, numerous genotype–diet interactions were found when fish were fed with or without fishmeal. As we know that plants are naturally rich in carbohydrates and rainbow trout are a typically glucose-intolerant fish, we hypothesized genotype–dietary carbohydrates interactions using these fish lines.

In this context, the present study aims to investigate how glucose metabolism could be affected by carbohydrate intake alone. The second objective is also to compare the putative differences of dietary carbohydrate utilization between the two isogenic lines, A32h and AB1h, in order to provide scientific support for the existence of genetic variability. To do so, we compared growth performance, whole body composition, plasma metabolites (glucose and triglycerides), hepatic nutrient sensing (linked to insulin signalling pathway using protein kinase B called Akt), amino acid sensor (using p70 S6 kinase, S6k) and energy/glucose sensors (using AMP-activated protein kinase, Ampk) (Kamalam et al., 2012; Dai et al., 2013) and numerous metabolic gene expressions (including the paralogs) associated with glucose use and that we know to be highly regulated at the molecular level (see Kamalam et al., 2017 for review).

## RESULTS

### Growth performance and whole body composition

After 12 weeks of feeding with or without carbohydrates, the zootechnical parameters of the two isogenic fish lines were obtained. For all of the studied parameters; final body weight (FBW), feed intake (FI), special growth rate (SGR), feed efficiency (FE), and protein retention efficiency (PRE), there were significant differences between the two lines (Table 1, *P*<0.05), for example, AB1h had lower FE and PRE than A32h (*P*<0.05). Moreover, FBW, FI and SGR were also significantly different between dietary treatments (Table 1, *P*<0.05). Interestingly, significant interactions were found for some of the previous parameters: only AB1h had higher FBW, FI and SGR when fed a high-carbohydrate (H-CHO) diet (Table 1, *P*<0.05).

**Table 1.**
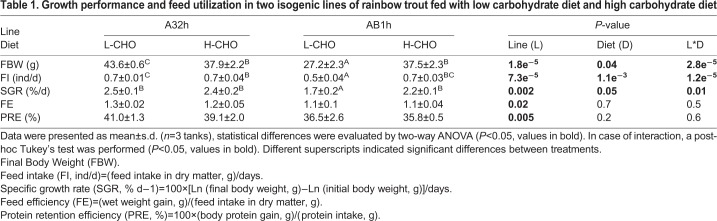
**Growth performance and feed utilization in two isogenic lines of rainbow trout fed with low carbohydrate diet and high carbohydrate diet**

Whole body compositions are shown in Table 2. Fish fed the H-CHO diet had a higher body lipid content (and thus also higher energy content) than the ones fed a low-carbohydrate (L-CHO) diet. Moreover, AB1h had higher lipid and energy contents than A32h (*P*<0.05). In contrast, no significant effect on whole body protein content was found. Finally, no interactions between lines and diets were detected for whole body composition.

**Table 2.**
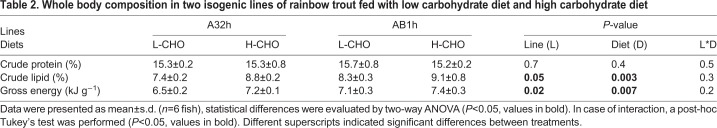
**Whole body composition in two isogenic lines of rainbow trout fed with low carbohydrate diet and high carbohydrate diet**

### Postprandial plasma metabolite levels

Plasma concentrations of specific metabolites (glucose and triglyceride) measured at 6 h after the last meal are presented in Table 3. Higher plasma glucose was observed in fish fed with the H-CHO diet (*P*<0.05). Higher plasma triglyceride was also detected in fish fed the H-CHO diet (*P*<0.05) and also in the AB1h isogenic line (*P*<0.05).

**Table 3.**
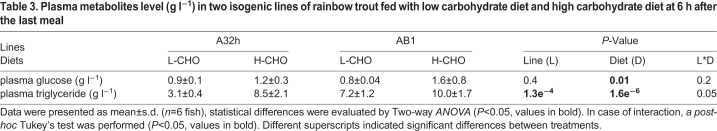
**Plasma metabolites level (g l^−1^) in two isogenic lines of rainbow trout fed with low carbohydrate diet and high carbohydrate diet at 6 h after the last meal**

### Nutrient and endocrinal sensing in the liver

**Fig. 1.**
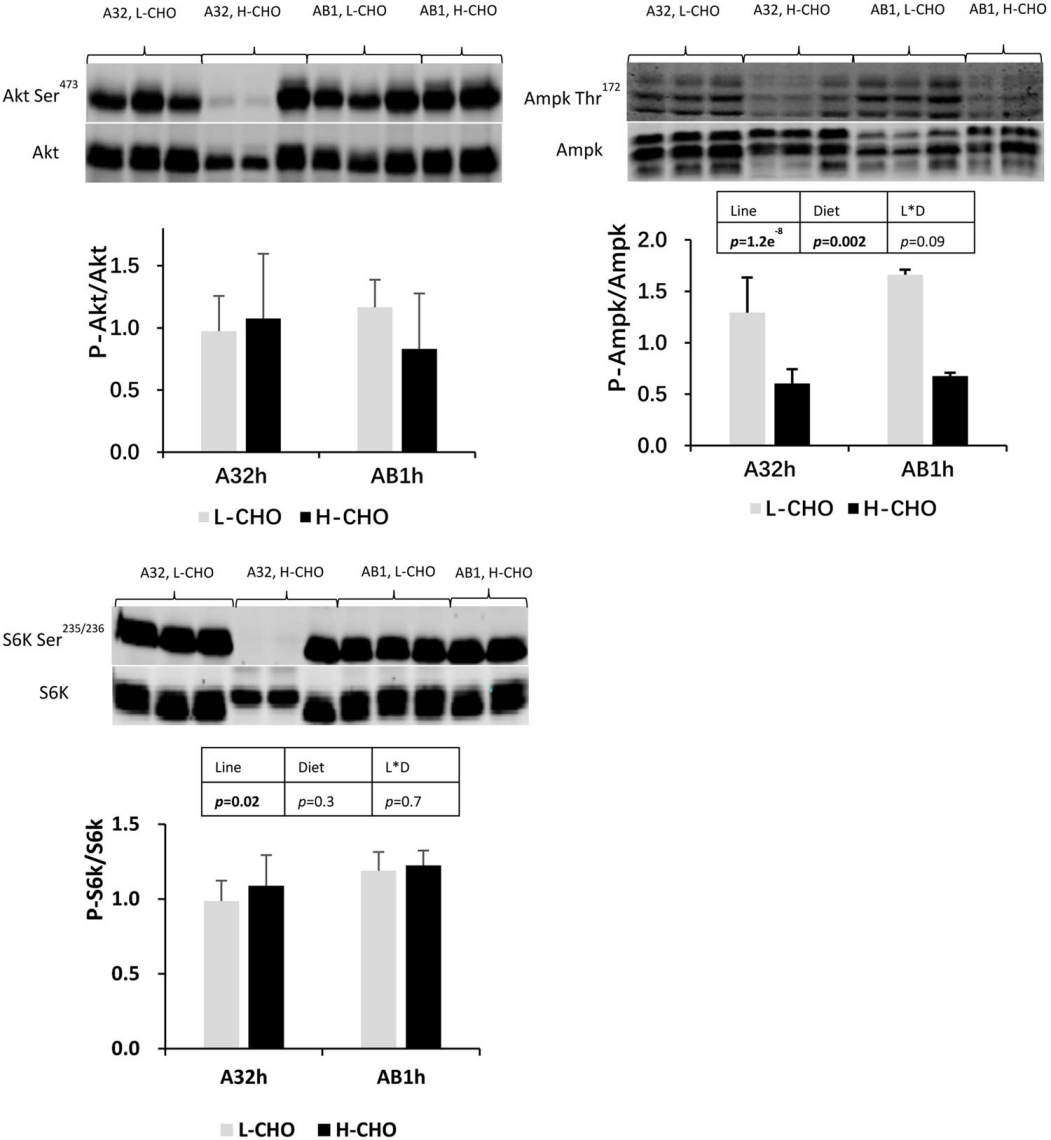
**Analysis of hepatic Akt, Ampk and S6k protein phosphorylation in two lines of rainbow trout fed with low carbohydrate diet and high carbohydrate diet.** Akt, protein kinase B; Ampk, 5′ adenosine monophosphate activated protein kinase; S6k, ribosomal protein S6 kinase. A representative blot is shown. Data were presented as mean±s.d. (*n*=6), the statistical differences of Akt, Ampk and S6k were evaluated by two-way ANOVA (*P*<0.05, values in bold).

In order to investigate the effects of dietary carbohydrates on the regulation of nutrient sensing (hormonal, amino acids and energy sensors) in trout liver, we analysed the phosphorylation status of Akt, Ampk and S6k proteins in the liver of trout lines at 6 h after the last meal (Fig. 1). No diet effect or genotype effect was observed on hepatic Akt protein. The phosphorylation of hepatic Ampk was significantly decreased in fish fed with the H-CHO diet (*P*<0.05). Finally, Ampk and S6k phosphorylation were higher in AB1h compared to A32h (*P*<0.05).

### Glucose metabolism in the liver

**Fig. 2.**
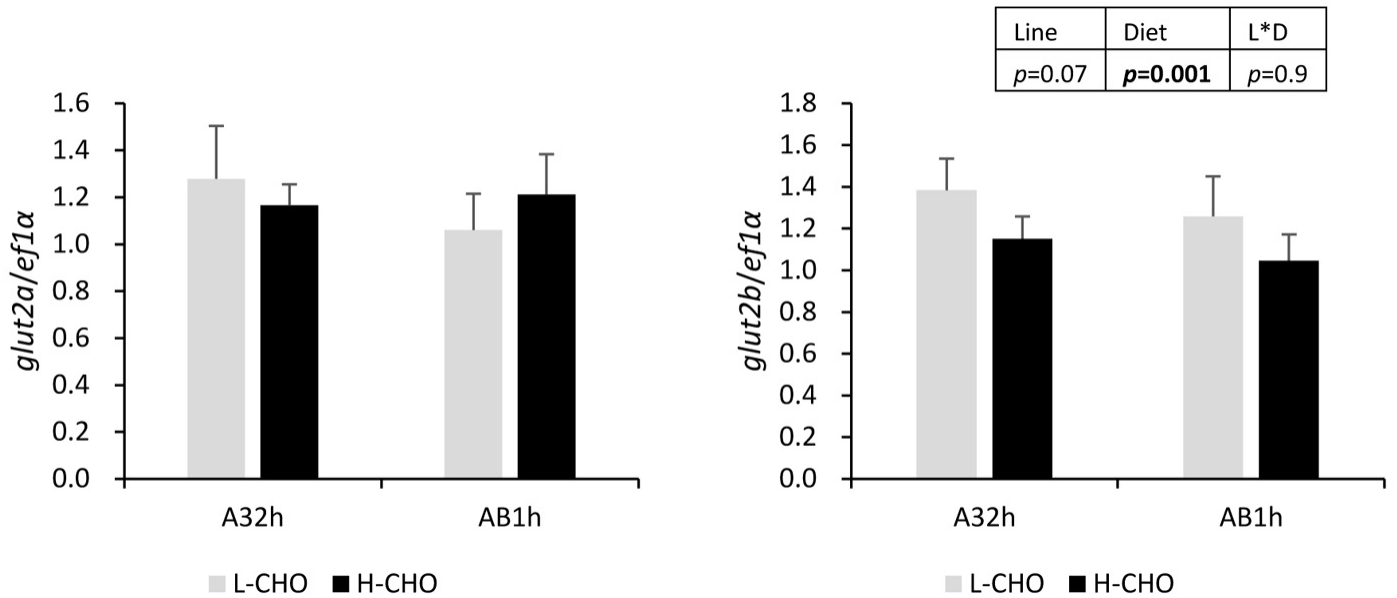
**mRNA levels of selected glucose transporter in the liver of two lines of rainbow trout fed with low carbohydrate diet and high carbohydrate diet.** *Glut2a* and *glut2b*, glucose transporter 2 paralogs. Data were presented as mean±s.d. (*n*=6), the statistical differences of *glut2a* and *gut2b* were evaluated by two-way ANOVA (*P*<0.05, values in bold).

As the first actor involved in glucose transport in and out of the liver, we studied the gene coding for the Glut2 protein. No difference in the expression of *glut2a* was found between lines and diets (*P*>0.05). However, *glut2b* was expressed at a lower level in trout fed with the H-CHO diet (Fig. 2, *P*<0.05).

**Fig. 3.**
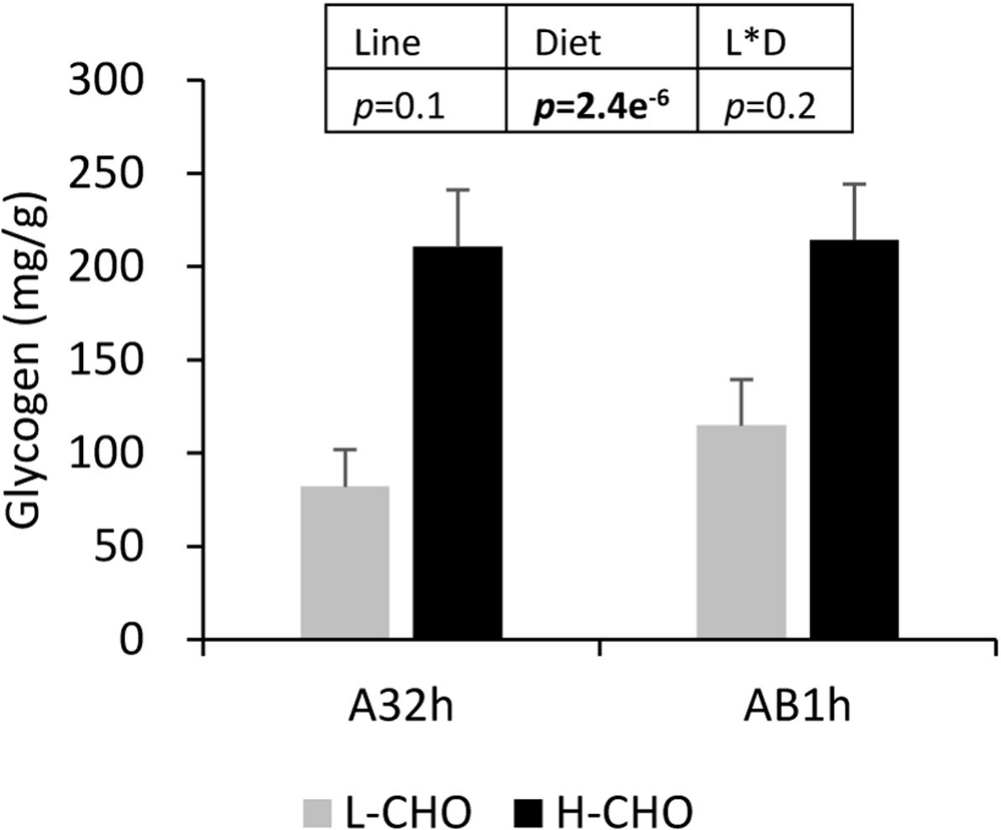
**Liver glycogen in two lines of rainbow trout fed with low carbohydrate diet and high carbohydrate diet.** Data were presented as mean±s.d. (*n*=6), statistical difference of liver glycogen was evaluated by two-way ANOVA (*P*<0.05, values in bold).

After transport inside the liver, excess glucose will be stored in the form of glycogen. Liver glycogen contents at 6 h after the last meal is shown in Fig. 3. Liver glycogen was significantly increased in fish fed with the H-CHO diet (*P*<0.05), but no differences related to lines was observed.

**Fig. 4.**
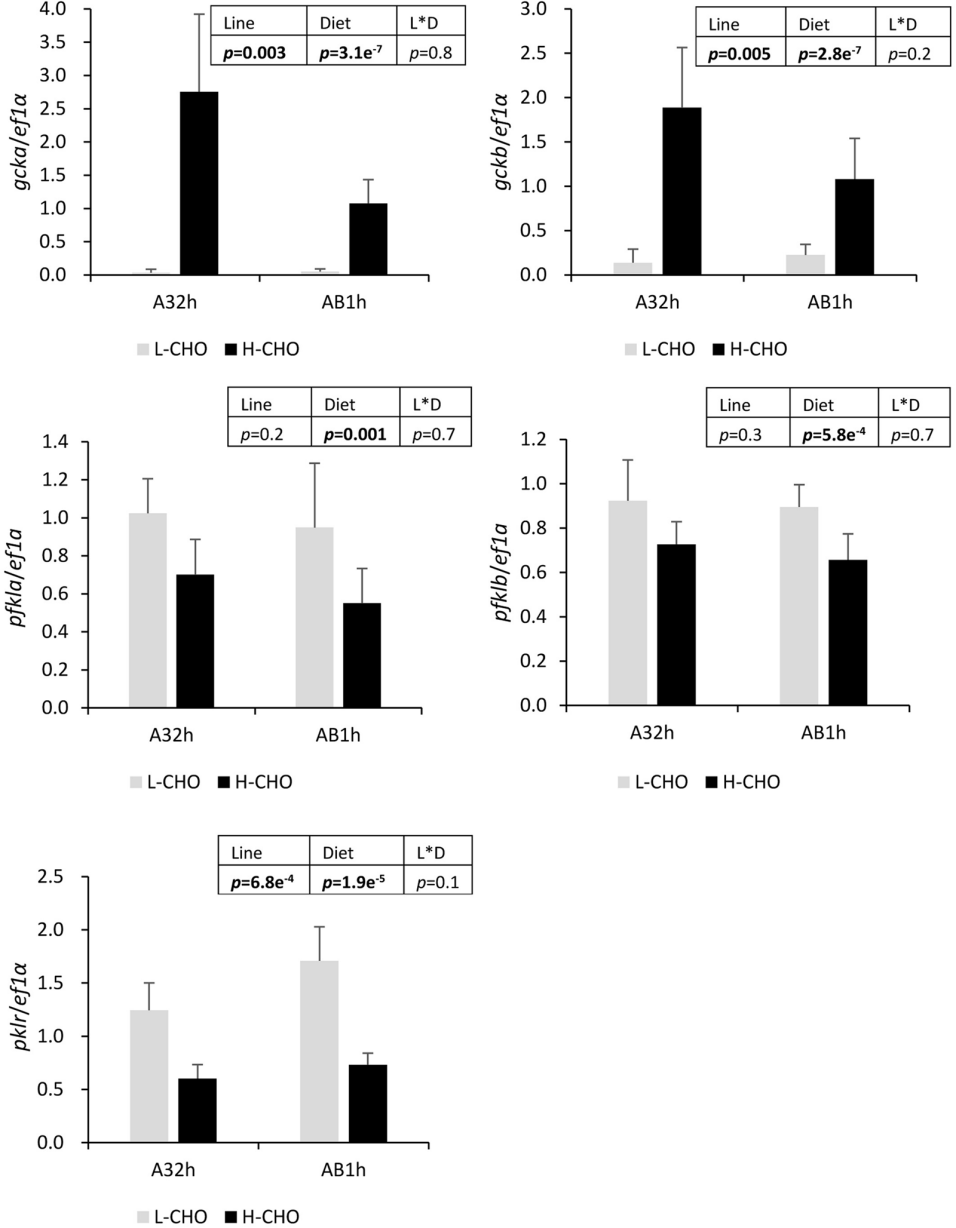
**mRNA levels of selected glycotic enzymes in the liver of two lines of rainbow trout fed with low carbohydrate diet and high carbohydrate diet.** *Gcka* and *gckb*, glucokinase paralogs; *pfkl*, 6-phosphofructokinase, liver type; *pkl*, pyruvate kinase, liver type. Data were presented as mean±s.d. (*n*=6), the statistical differences of g*cka, gckb, pfkl* and *pkl* were evaluated by two-way ANOVA (*P*<0.05, values in bold).

Regarding the glycolysis in the liver, the expression of target genes encoding key enzymes were analysed (Fig. 4). The mRNA levels of *gcka* and *gckb* were markedly higher in fish fed the H-CHO diet, and AB1h showed lower mRNA levels of *gcka* and *gckb* than A32h (*P*<0.05). However, lower *pfkla, pfklb* and *pklr* mRNA levels were found in trout fed with the H-CHO diet. Moreover, *pklr* mRNA levels were also higher in AB1h than in A32h (*P*<0.05).

**Fig. 5.**
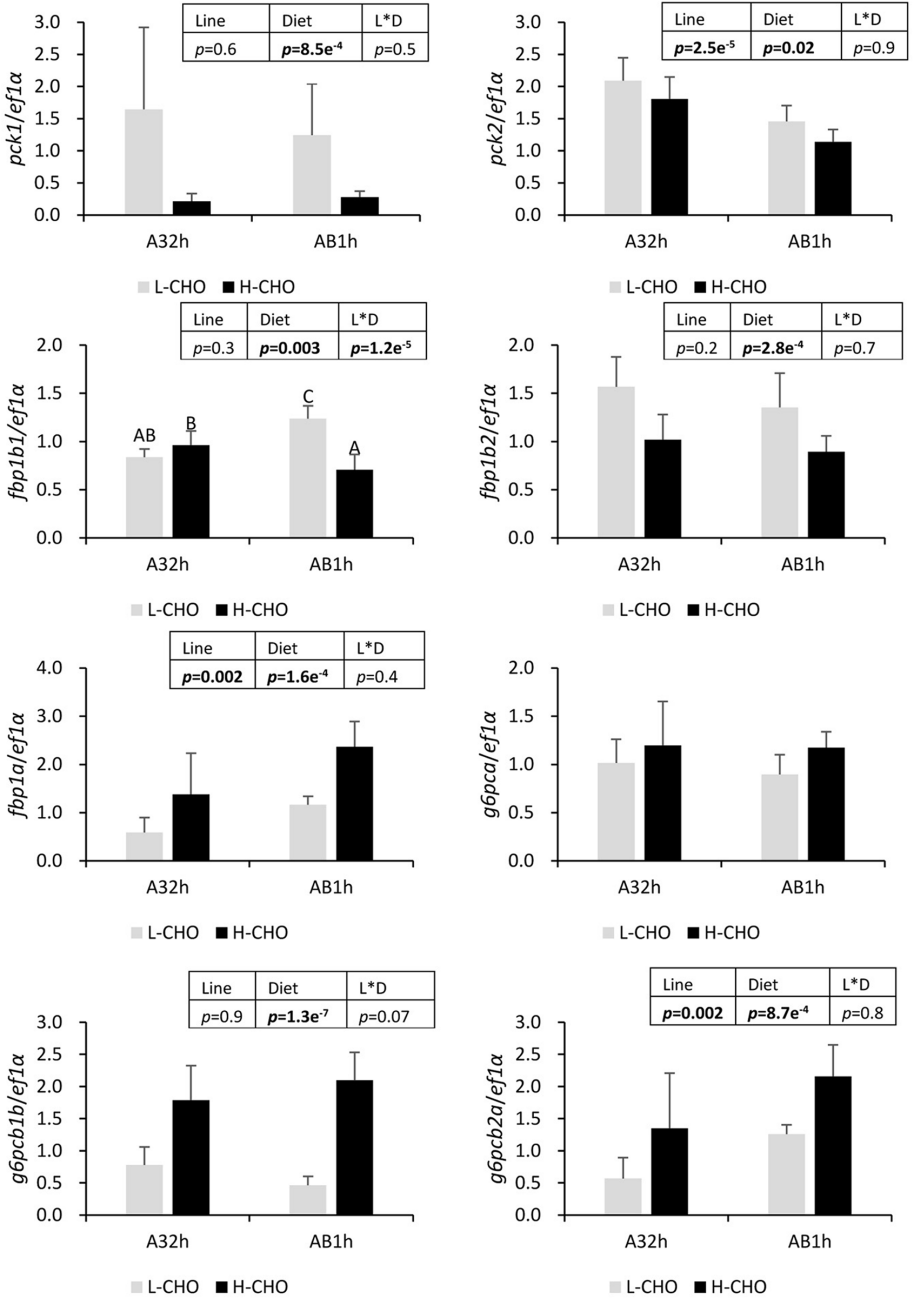
**mRNA levels of selected gluconeogenesis enzymes in the liver of two lines of rainbow trout fed with low carbohydrate diet and high carbohydrate diet.** *pck1* and *pck2*, phosphoenolpyruvate carboxykinase paralogs. *fbp1b1*, *fbp1b2* and *fbp1a*, fructose 1,6-bisphosphatase paralogs; *g6pca*, *g6pcb1b* and *g6pcb2a*, glucose 6-phosphatase paralogs. Data were presented as mean±s.d. (*n*=6), the statistical differences of *pck1*, *pck2*, *fbp1b2*, *fbp1a*, *g6pca*, *g6pcb1b* and *g6pcb2a* were evaluated by two-way ANOVA (*P*<0.05, values in bold). Because of interaction, a post-hoc Tukey's test was performed for *fbp1b1*. Different superscripts indicated significant differences between treatments.

Gluconeogenesis, the pathway involved in hepatic glucose production, was also studied through the analysis of key gluconeogenic enzymes (Fig. 5). When fed with the H-CHO diet, trout exhibited a significant decrease in the mRNA levels of *pck1*, *pck2* and *fbp1b2* but also showed a significant increase in the mRNA levels of *fbp1a*, *g6pcb1b* and *g6pcb2a* (*P*<0.05). In regards to the effect of isogenic lines, AB1h displayed a lower mRNA level of *pck2* than A32h, whereas mRNA levels of *fbp1a* and *g6pcb2a* were higher in AB1h than in A32h (*P*<0.05). A significant line×diet interaction in the mRNA levels of *fbp1b1* was also detected, a decrease of *fbp1b1* with the H-CHO diet was only observed in AB1h (*P*<0.05). Finally, no detectable mRNA levels for *g6pcb1a* and *g6pcb2b* paralogues were detected.

### Bioconversion of excess glucose to fatty acids: lipogenic gene expression in liver

**Fig. 6.**
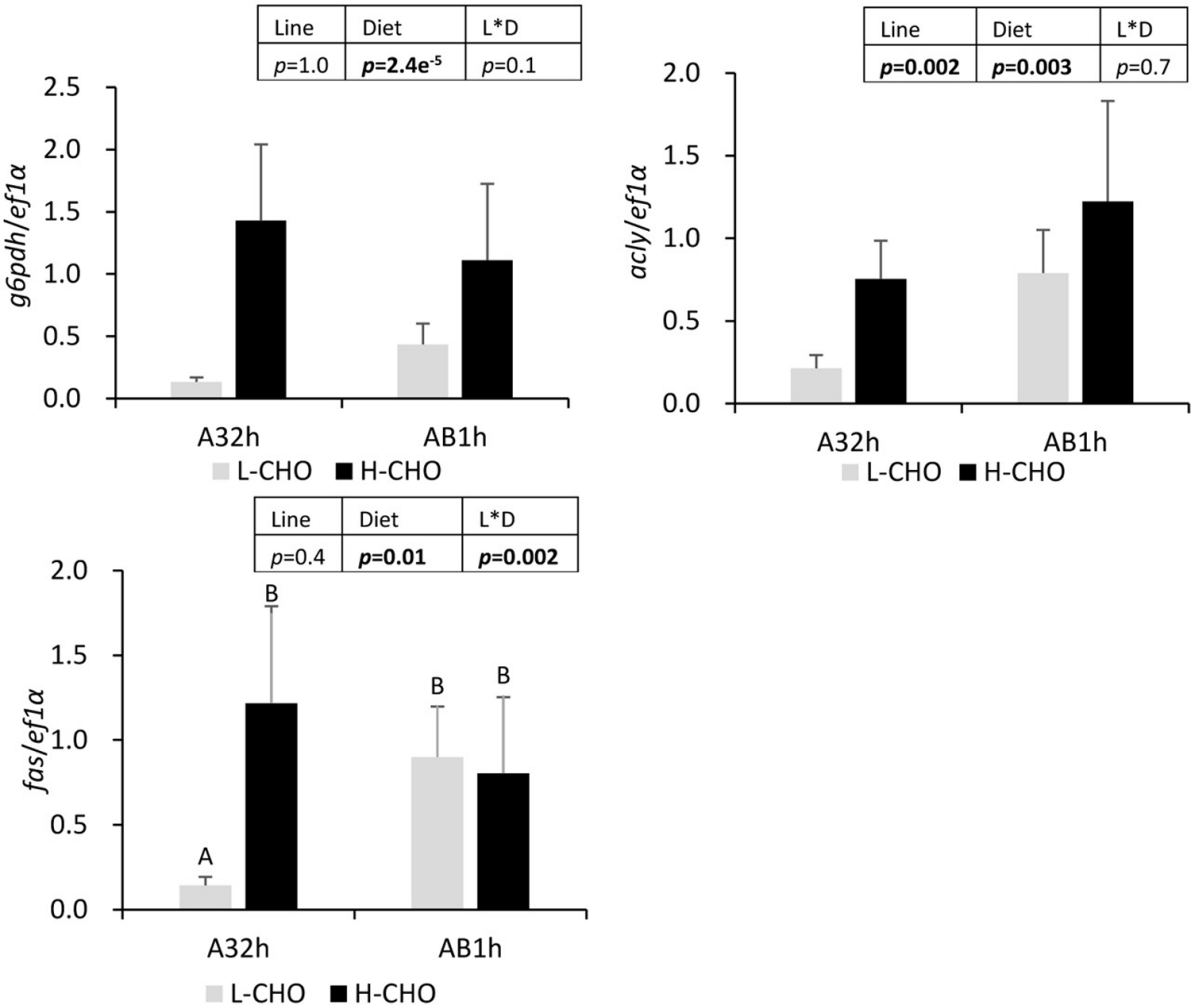
**mRNA levels of selected lipogenesis enzymes in the liver of two lines of rainbow trout fed with low carbohydrate diet and high carbohydrate diet.** *G6pcdh*, glucose 6-phosphate dehydrogenase; *acly*, adenosine triphosphate citrate lyase; *fas*, fatty acid synthase. Data were presented as mean±s.d. (*n*=6), the statistical differences of *g6pcdh* and *acly* were evaluated by two-way ANOVA (*P*<0.05, values in bold). Because of interaction, a post-hoc Tukey's test was performed for *fas*. Different superscripts indicated significant differences between treatments.

We further measured the expressions of selected enzymes involved in fatty acid biosynthesis in the liver (Fig. 6). A high carbohydrate diet increased mRNA levels of *g6pdh* and *acly*. AB1h showed a significantly higher mRNA level of *acly* than A32h (*P*<0.05). There was a significant line×diet interaction in the mRNA levels of *fas*, and A32h fed with the low carbohydrate diet showed lower expression of *fas* compared with other groups (*P*<0.05).

## DISCUSSION

Rainbow trout are a representative carnivorous fish (NRC, 2011). As described by Hemre et al. (2002), levels of more than 20% of dietary carbohydrates in feed decrease growth performance and cause metabolic disturbance in salmonids. Indeed, it is well known that carnivorous fish, such as rainbow trout, fed with a high level of digestible carbohydrates are hyperglycaemic (Polakof et al., 2012; Kamalam et al., 2017). Even though some hypotheses linked to a dysfunction of the liver have been previously described (Polakof et al., 2012; Kamalam et al., 2017), some questions remain: (1) all the previous analyses to test the effects of carbohydrates in trout were performed using different carbohydrates/proteins ratios and the questions about the potential effects of proteins on the regulation of the glucose metabolism is still under debate (Seiliez et al., 2011; Skiba-Cassy et al., 2013; Dai et al., 2013) (2) the existence or not of differences in the dietary glucose metabolism in trout between different genotypes. To answer these two questions, we fed two isogenic lines (all individuals of each line sharing the same genotype) with (23%) or without (3.6%) digestible carbohydrates but with the same level of dietary proteins (48%).

### Digestible dietary carbohydrates strongly affect hepatic glucose metabolism at the molecular level in rainbow trout

Regarding the growth performance, inclusion of digestible dietary carbohydrates impacted the FBW, FI and SGR but this differed between the fish lines (see the last paragraph of the Discussion). On the other hand, we did not find any differences in FE and PRE, which currently shows the absence of a protein-sparing effect of digestible carbohydrates in contrast to what was observed when the dietary protein/carbohydrate ratios were modified (Kaushik et al., 1989; Kim and Kaushik, 1992; Wilson, 1994).

The main objective of this study was to analyse the regulation of glucose metabolism by dietary carbohydrates alone. In the present study, although the increase of glycaemia found in fish fed with the high carbohydrate diet was as expected (1.2 g/l or 1.6 g/l in the two lines fed H-CHO), the increase of glycaemia was much more moderate compared to previous studies (Wilson, 1994; Hemre et al., 2002; Panserat et al., 2009). This relatively low level of postprandial glycaemia could be related to the low level of dietary lipids in the two diets, as observed previously (Figueiredo-Silva et al., 2012).

We also analysed the liver as the key organ for intermediary metabolism and its regulation of glucose homeostasis. The first step is glucose transport in the liver which is carried out by glucose transporter 2. Our data showed that the *glut2b* mRNA level was lower in fish fed with carbohydrates, which is particularly surprising because this gene is not strongly regulated by dietary glucose (Kirchner et al., 2008; Polakof et al., 2010; Jin et al., 2014). Moreover, we also studied the nutrient sensing pathway (Ampk for energy sensing, Akt for insulin signalling, S6k for mTOR and amino acid sensing) which is important for the regulation of metabolic pathways in the liver. Firstly, phosphorylated Ampk [a metabolic master by which cells sense and decode changes in cellular energy status (Zhang et al., 2009) but which has also recently been determined as a glucose sensor (Lin and Hardie, 2017)] is dramatically decreased in fish fed with digestible carbohydrates as expected and as shown previously by Kamalam et al. (2012). By contrast, Akt and S6k proteins were not affected by dietary carbohydrate content, suggesting that insulin and mTOR signalling pathways were not changed. Knowing that insulin secretion does not seem to depend on glucose but on amino acids in trout (Mommsen and Plisetkaya, 1991; Panserat et al., 2000b), the same level of dietary proteins in both diets probably induced the same level of insulinemia and aminoacidemia and may thus explain the absence of variation of the insulin and mTOR signalling pathways.

We analysed the molecular regulation of glucose metabolism in the liver by examining the regulation of glycolysis and gluconeogenesis. After ingestion of carbohydrates, in order to avoid hyperglycaemia, glycolysis is induced and gluconeogenesis reduced in liver (van de Werve et al., 2000; Iynedjian, 2009). Glucokinase is the first key enzyme which phosphorylates glucose into glucose-6-phosphate. It had been discovered that the *gck* gene is highly induced by H-CHO diet in different fish species (Panserat et al., 2000a; Panserat et al., 2014). Indeed, in our study, both *gcka* and *gckb* paralogs were dramatically increased in trout fed with a high carbohydrate diet as previously observed (Marandel et al., 2016). However, the mRNAs *pfkla*, *pfklb* and *pklr* genes encoding for the other two key glycolytic enzymes were surprisingly less expressed in fish fed with H-CHO diet than in fish fed with L-CHO diet. This counter-intuitive regulation of two key glycolytic enzymes can be associated with a poor glycaemia control. Regarding the gluconeogenesis (and the hepatic glucose production), some of the gluconeogenic genes (*pck1*, *fpb1b2*) were less expressed in fish fed with the H-CHO diet, some were not regulated (*pck2*, *g6pca*) but others were more highly expressed (*fpb1a*, *g6pcb1b* and *g6pcb2a*). Our data confirmed that hepatic gluconeogenesis is poorly regulated by dietary carbohydrates in trout (Polakof et al., 2012), in particular it is linked to the specific regulation of *g6pc* duplicated genes (Marandel et al., 2015, 2016). All our data reinforces the hypothesis that the non-induction of some glycolytic genes and the non-inhibition of some gluconeogenic genes may be involved in the establishment of the glucose-intolerant phenotype in rainbow trout.

Excess glucose (after ingesting carbohydrates) has to be stored as glycogen and/or converted into triglycerides through lipogenesis (Towle et al., 1997; Skiba-Cassy et al., 2009; Polakof et al., 2012). Our data confirmed that trout fed with the H-CHO diet had higher hepatic glycogen and higher gene expression for enzymes involved in lipogenesis (*acly* and *g6pd*) associated with higher plasma triglycerides and fat content in the whole body.

### Two divergent isogenic lines for growth performance and hepatic intermediary metabolism

The objective of our study was to investigate possible differences in the nutritional and metabolic responses to carbohydrate-rich diets according to genetic background (i.e. the two isogenic lines, A32h and AB1h). Our data demonstrated that AB1h showed lower growth performance (FBW and SGR) than A32h, probably linked to the significantly lower FI. Moreover, AB1h presented lower FE and PER than A32h which can also explain the lower growth performance.

Even though no differences in postprandial glycaemia and hepatic glycogen were observed between the two lines fed with the two diets, some differences appeared at the level of hepatic glucose metabolism. Indeed, lower *gcka* and *gckb* mRNA levels were observed in AB1h line whereas mRNA levels of genes encoding for the other two glycolytic enzymes were higher, which could be linked to better hepatic glycolysis in AB1h. Regarding the gluconeogenesis, lower *pck2* mRNA levels but higher *fbp1a* and *g6pcb2a* mRNA levels were also observed in the AB1h line, which could be associated with higher hepatic glucose production in AB1h. Although it is difficult to conclude that AB1h has a better metabolic ability to use dietary glucose than A32h, our data clearly shows the existence of differences in molecular regulation of glucose metabolism between the two trout genotypes, as previously observed in fat/lean lines (Kamalam et al., 2012; Jin et al., 2014). Indeed, it also must be noted that phosphorylation levels of Ampk and S6k are higher in AB1h, suggesting differences in nutrient sensing capacity between the two lines and confirming that intermediary metabolism could be differently regulated between the two lines.

Regarding lipid metabolism, our data showed higher whole-body lipid and energy content in AB1h, associated with a higher level of plasma triglycerides and a higher *acly* gene mRNA level. It seems that AB1h have a higher capacity to store and/or produce lipids. This could explain the lower FE and PER in AB1h knowing that the production of lipids is ATP/energy consuming (Kolditz et al., 2008; Nguyen et al., 2008). Moreover, because poor lipogenic capacity can be one of the hypotheses for a poor dietary glucose use in trout (Polakof et al., 2012; Kamalam et al., 2017), our data could suggest that AB1h could have a higher potential of dietary glucose use.

### Are isogenic lines differently used dietary carbohydrates?

Only a few interactions between the diets and the genotypes (lines) were found in the present experiment (despite the important interactions seen for the FBW, SGR and FI parameters), as previously observed in some rainbow trout lines selected on fat muscle content (Skiba-Cassy et al., 2009). Indeed, AB1h fed the L-CHO diet had lower growth performance than A32h, whereas AB1h fed the H-CHO carbohydrate had the same growth performance as A32h. Unfortunately, the strong interactions for the growth performance did not seem to be clearly linked to variations of the hepatic intermediary metabolism, except for the *fpb1b1* gene which was down regulated by the H-CHO diet only in the AB1h line, and the *fas* gene mRNA level which was up-regulated by the H-CHO diet only in the A32h line. These molecular results do not show strong evidence for the preferable ability of using carbohydrate in AB1h, therefore we speculated that the inclusion of dietary digestible carbohydrates was important for better growth only for AB1h, and was probably linked to the decrease in feed intake in AB1h fed high level of cellulose. No clear conclusion can be formulated regarding the existence of one fish line showing better use of carbohydrates at a metabolic level.

### Conclusion

In summary, our experiment demonstrated for the first time the existence of an atypical regulation by carbohydrates of some of the genes involved in glycolysis and gluconeogenesis in the liver. These findings may explain, in part, the poor metabolic carbohydrate use in rainbow trout. Although we cannot conclude which line (A32h or AB1h) had overwhelming superiority in their positive response to a high carbohydrate diet, there were numerous differences in growth performance and molecular metabolism between the two lines. Globally, our results provide key support for future specific selection, especially for carbohydrate-tolerant trout breeding. Finally, our data also confirmed that the isogenic lines are powerful tools to continue research about nutrition genetic interactions.

## MATERIALS AND METHODS

### Ethical statement

Experimentation was conducted in the INRA experimental facilities (Donzacq, UMR Numéa, St-Pée-sur-Nivelle, France) authorized for animal experimentation by the French veterinary service which is the competent authority (A 64-495-1). The experiments were in strict accordance with EU legal frameworks related to the protection of animals used for scientific research (Directive 2010/63/EU) and according to the National Guidelines for Animal Care of the French Ministry of Research (decree n°2013-118, February 1st, 2013). In agreement with ethical committee ‘comité d’éthique Aquitain poissons oiseaux’ (C2EA-73), the experiment reported here does not need approval by a specific ethical committee since it implies only classical rearing practices with all diets used in the experimental formulated to cover all the nutritional requirements of rainbow trout (NRC, 2011).

### Fish experimental diets

Two experimental diets for juvenile rainbow trout, called the L-CHO and H-CHO diets, were manufactured at INRA, Donzacq, Landes, France. They were formulated as extruded pellets to contain the same level of proteins and lipids, but different levels of carbohydrates, as shown in Table 4. Dietary protein (∼48%) was provided via fishmeal and soluble fish protein concentrate, dietary lipid (∼8%–11%) was provided via fish oil and fishmeal, gelatinized starch was included as the digestible carbohydrate source [3.6% (L-CHO) and 22.9% (H-CHO) respectively]. The digestible gelatinized starch in the H-CHO diet was compensated for with non-digestible cellulose in the L-CHO diet, which implied a difference in digestible energy content between both lines.

**Table 4.**
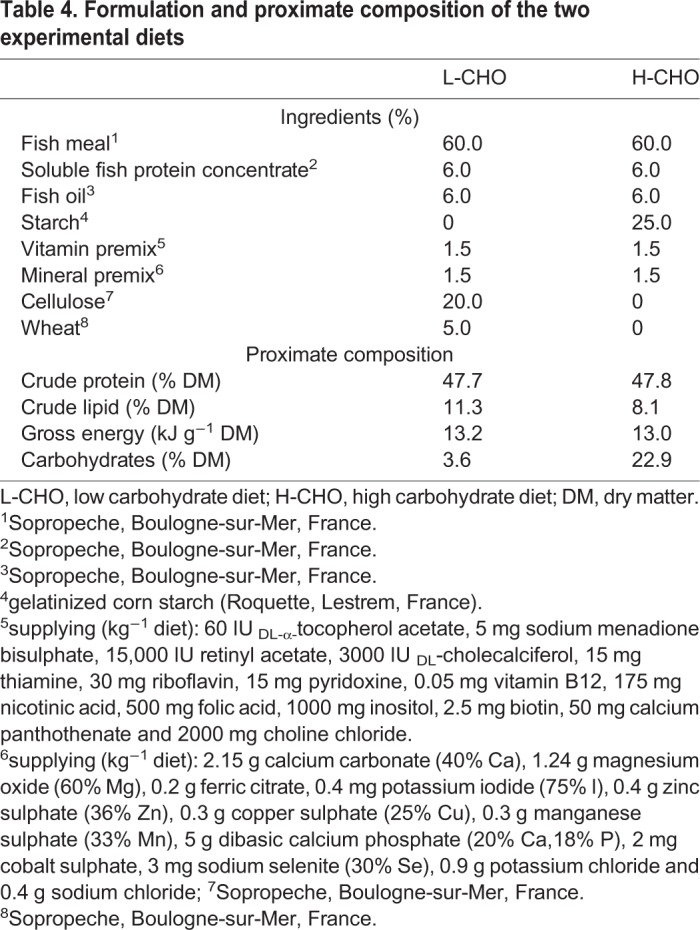
**Formulation and proximate composition of the two experimental diets**

### Isogenic fish, nutritional experiment and sampling procedure

Two heterozygous isogenic lines of rainbow trout (*O.* *mykiss*) were obtained by mating sires and dams from homozygous isogenic lines (Peima, Sizun, France). The homozygous lines used as broodstock had been previously established after two generations of gynogenesis and then maintained within lines by single-pair mating using sex reversed XX males (Quillet et al., 2007). Eggs from fully homozygous females from line B57 were mixed and fertilized in two separated batches with milt from two homozygous sires from A32 and AB1 lines to produce heterozygous lines named as A32h and AB1h. Therefore, genetic differences between the two lines could be attributed only to paternal genetic differences, and all individuals within one line shared the same genotype.

Rainbow trout were reared at 18°C in the INRA experimental facilities at Donzacq, Landes, France, under a natural photoperiod. Fish of each line (∼5 g) (A32h and AB1h) were randomly distributed into tanks at the density of 20 fish/tank, each line being fed with L-CHO or H-CHO. Fish were reared in triplicates for all the experimental conditions (two lines×two diets in triplicate i.e. *n*=12 tanks in total). All fish were fed twice a day to apparent satiation. After 12 weeks of feeding, two fish per tank were randomly sampled at 6 h after the last meal, known to be the peak of postprandial glycaemia in rainbow trout fed with carbohydrates at 18°C (Polakof et al., 2012; Kamalam et al., 2017). Trout were anesthetized with benzocaine (30 mg/l) and killed by a sharp blow to the head. Blood was removed from the caudal vein via heparinized syringes and centrifuged (3000 ***g***, 5 min). The plasma recovered was immediately frozen and kept at –20°C until analysis. The fresh liver was collected and immediately frozen in liquid nitrogen and then kept at –80°C. Later, six more fish per tank were randomly sampled at 48 h after the last meal. They were immediately frozen and kept at –20°C for whole-body composition determination.

### Chemical analysis for diets and whole body composition

The chemical composition of diets and fish were analysed by the following procedures: protein content (*N*×6.25) was determined by using the Kjeldahl method after acid digestion; fat was determined by petroleum ether extraction (Soxtherm, Konigswinter, Germany); gross energy was determined in an adiabatic bomb calorimeter (IKA, Heitersheim Gribheimer, Germany) and starch content was determined by an enzymatic method (InVivo Labs, Saint Nolff, France).

### Metabolite analysis in the plasma and liver

Plasma glucose and triglycerides were determined by using a commercial kit (Biomerieux, Marcy I'Etoile, France) adapted to microplate format according to the manufacturer's instructions. Liver glycogen was determined by a hydrolysis technique previously described by Good et al. (1933). Each sample was mixed in 1 mol^−1^ HCL (VWR, Radnor, USA). An aliquot (200 μl) was saved at this stage to measure the free glucose content after 10 min centrifugation at 10,000 ***g***, measured using the Amplite™, Fluorimetric Glucose Quantitation Kit (AAT Bioquest^®^ Inc., Sunnyvale, USA) according to the manufacturer's instructions. The remaining ground tissue was boiled at 100°C for 2.5 h and then the pH was adjusted to 7.4 by neutralizing with 5 mol l^−1^KOH (VWR). Total glucose (free glucose+glucose obtained from glycogen hydrolysis) was measured using the same kit as before. Glycogen content was evaluated by subtracting free glucose levels.

### Western blot analysis

Frozen livers (70–100 mg) were weighed into 2 ml of lysis buffer [150 mM NaCl, 10 mM Tris, 1 mM EGTA, 1 mM EDTA (pH 7.4), 100 mM sodium fluoride, 4 mM sodium pyrophosphate, 2 mM sodium orthovanadate, 1% Triton X-100, 0.5% NP-40-IGEPAL, and a protease inhibitor cocktail (Roche, Basel, Switzerland)] and homogenized on ice using ULTRA-TURRAX homogenizer (IKA-WERKE, Staufen im Breisgau, Germany). Homogenates were centrifuged at 1000 ***g*** for 15 min at 4°C, and then we recovered the supernatant to centrifuge again at 2000 ***g*** for 30 min at 4°C. The resulting supernatant fractions were obtained and stored at –80°C. Protein concentrations were determined using the Bio-Rad protein assay kit (Bio-Rad Laboratories, Munich, Germany) with BSA (bovine serum albumin) as the standard. Lysates (20.3 μg of the total protein) were subjected to SDS-PAGE. Appropriate antibodies were obtained from Cell Signaling Technology. Anti-phospho-Akt (Ser^473^) (no.4060), anti-Akt (no.9272), Anti-phospho-Ampk (Thr^172^) (no.2531), anti-Ampk (no.2532), Anti-phospho-S6k (Ser^235/236^) (no.4856), anti-S6k (no.2217) were used on the western blots. All the antibodies have been shown to cross react successfully with rainbow trout proteins of interest (Dai et al., 2013; Jin et al., 2014). After washing, membranes were incubated with an IRDye infrared secondary antibody (LI-COR Biosciences, Lincoln, USA). The bands were visualized by infrared fluorescence using the odyssey Imaging System (LI-COR Biosciences) and quantified by odyssey infrared Imaging System software (version 3.0, LI-COR Biosciences).

### mRNA-levels analysis

Total-RNA samples were conducted on the liver. Samples were extracted using TRIzol reagent (Invitrogen), according to the manufacturer's recommendations and were quantified by spectrophotometry (absorbance at 260 nm). The integrity of the samples was assessed using agarose-gel electrophoresis. 1 μg of total RNA per sample was reverse transcribed into cDNA using the SuperScript III reverse transcriptase kit (Invitrogen) with random primers (Promega, Charbonnieres, France) according to the manufacturer's instructions.

mRNA levels of key target glucose metabolic genes were determined by quantitative real-time (q) RT-PCR. Elongation factor-1 alpha (*ef1α*) was regarded as the reference gene which was stably expressed in the studies of Olsvik et al. (2005) and the primers of target genes had already been published in previous studies (Marandel et al., 2015, 2016; Liu et al., 2017) using specific primers for the paralogs shown in Table 5. In the present study we analysed the mRNA levels of genes encoding glycolytic enzymes (*gck* coding the glucokinase, EC 2.7.1.2; *pfkl* coding for the 6-phosphofructokinase, EC 2.7.1.11; *pkl* coding the pyruvate kinase, EC. 2.7.1.40) and gluconeogenesis (*pck* coding for the phosphoenolpyruvate carboxykinase, EC 4.1.1.32; *fbp* coding for the fructose 1,6-bisphosphatase, EC 3.1.3.11; *g6pc* coding for the glucose 6-phosphatase, EC 3.1.3.9) and lipogenesis (*G6pdh* coding the glucose 6-phosphate dehydrogenase, EC 1.1.1.49; *acly* coding the adenosine triphosphate citrate lyase, EC 2.3.3.8; *fas* coding the fatty acid synthase, EC 2.3.1.85). Quantitative RT-PCRs were carried out on a Light Cycle 480 II (Roche Diagnostics, Neuilly-sur-Seine, France) using SYBR Green I Master (Roche Diagnostics). PCR was performed using 2 μl of the diluted cDNA (76 times) mixed with 0.24 μl of each primer (10 μM), 3 μl of Light Cycle 480 SYBR Green I Master (Roche Diagnostics) and 0.52 μl of DNase/RNase/protease-free water (5 prime, Hamburg, Germany) in a total volume of 6 μl. The qPCR was initiated at 95°C for 10 min, then followed by 45 cycles of a three-step amplification program (15 s at 95°C, 10 s at 60°C, 15 s at 72°C). Melting curves were systematically monitored (5 s at 95°C, 1 min at 65°C, temperature gradient 0.11°C/s from 65–97°C) at the end of the last amplification cycle to confirm the specificity of the amplification reaction. Each PCR assay included replicate samples (duplicate of reverse transcription and PCR amplification, respectively) and negative controls (reverse transcriptase and RNA-free samples). Relative quantification of target genes expression was performed using the E-Method from the Light Cycler 480 software (version SW 1.5; Roche Diagnostics). PCR efficiency was measured by the slope of a standard curve using serial dilution of cDNA, and it ranged between 1.90–2.0.

**Table 5.**
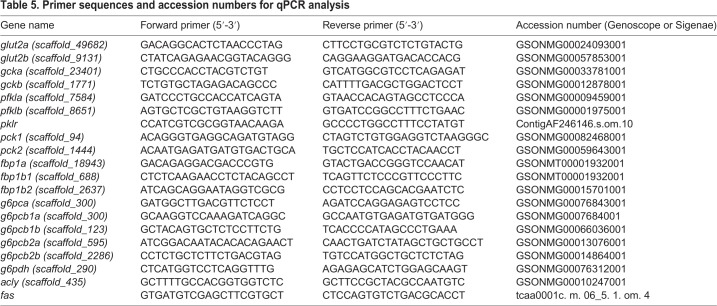
**Primer sequences and accession numbers for qPCR analysis**

### Statistical analysis

Normality of distributions was assessed by Shapiro-Wilk test. Data were analysed by Two-way ANOVA to assess the differences between lines, diets and interactions. If interactions between diets and lines were statistically significant, post-hoc Tukey test would be used to compare all the groups. Data were analysed with R software (v.3.3.3)/R Commander Package. Treatment effects and interactions were considered statistically significant at *P*<0.05. Results were presented as means±s.d. (*n*=6 samples per treatment based on non-significant differences between tanks per group).

## References

[BIO032896C1] Bergot F. (1979). Effects of dietary carbohydrates and of their mode of distribution on glycaemia in rainbow trout (*Salmo gairdneri* Richardson). *Comp. Biochem. Physiol.* 64A, 543-547. 10.1016/0300-9629(79)90581-4

[BIO032896C2] Berthelot C., Brunet F., Chalopin D., Juanchich A., Bernard M., Noël B., Bento P., Da Silva C., Labadie K., Alberti A. et al. (2014). The rainbow trout genome provides novel insights into evolution after whole-genome duplication in vertebrates. *Nat. Commun.* 5, 3657 10.1038/ncomms4657 24755649 PMC4071752

[BIO032896C3] Brauge C., Corraze G. and Médale F. (1995). Effects of dietary levels of carbohydrate and lipid on glucose oxidation and lipogenesis from glucose in rainbow trout, *Oncorhynchus mykiss*, reared in freshwater or in seawater. *Comp. Biochem. Physiol.* 111A, 117-124. 10.1016/0300-9629(95)98527-N

[BIO032896C4] Dai W., Panserat S., Mennigen J. A., Terrier F., Dias K., Seiliez I. and Skiba-Cassy S. (2013). Post-prandial regulation of hepatic glucokinase and lipogenesis requires the activation of TORC1 signalling in rainbow trout (*Oncorhynchus mykiss*). *J. Exp. Biol.* 216, 4483-4492. 10.1242/jeb.091157 24031053

[BIO032896C5] Dai W., Panserat S., Plagnes-Juan E., Seiliez I. and Skiba-Cassy S. (2015). Amino acids attenuate insulin action on gluconeogenesis and promote fatty acid biosynthesis via mTORC1 signaling pathway in trout hepatocytes. *Cell Physiol. Biochem.* 36, 1084-1100. 10.1159/000430281 26112996

[BIO032896C6] Dupont-Nivet M., Médale F., Leonard J., Le Guillou S., Tiquet F., Quillet E. and Geurden I. (2009). Evidence of genotype–diet interactions in the response of rainbow trout (*Oncorhynchus mykiss*) clones to a diet with or without fishmeal at early growth. *Aquaculture* 295, 15-21. 10.1016/j.aquaculture.2009.06.031

[BIO032896C7] Dupont-Nivet M., Robert-Granié C., Le Guillou S., Tiquet F. and Quillet E. (2012). Comparison of isogenic lines provides evidence that phenotypic plasticity is under genetic control in rainbow trout *Oncorhynchus mykiss*. *J. Fish Biol.* 81, 1754-1762. 10.1111/j.1095-8649.2012.03437.x 23020573

[BIO032896C8] Enes P., Panserat S., Kaushik S. and Oliva-Teles A. (2009). Nutritional regulation of hepatic glucose metabolism in fish. *Fish Physiol. Biochem.* 35, 519-539. 10.1007/s10695-008-9259-5 18791853

[BIO032896C9] Erfanullah, and Jafri A. K. (1995). Protein-sparing effect of dietary carbohydrate in diets for fingerling *Labeo rohita*. *Aquaculture* 136, 331-339. 10.1016/0044-8486(95)00056-9

[BIO032896C10] Figueiredo-Silva A. C., Panserat S., Kaushik S., Geurden I. and Polakof S. (2012). High levels of dietary fat impair glucose homeostasis in rainbow trout. *J. Exp. Biol.* 215, 169-178. 10.1242/jeb.063933 22162865

[BIO032896C11] Geurden I., Borchert P., Balasubramanian M. N., Schrama J. W., Dupont-Nivet M., Quillet E., Kaushik S. J., Panserat S. and Médale F. (2013). The positive impact of the early-feeding of a plant-based diet on its future acceptance and utilisation in rainbow trout. *PLoS ONE* 8, e83162 10.1371/journal.pone.0083162 24386155 PMC3873907

[BIO032896C12] Good C. A., Kramer H. and Somogyi M. (1933). The determination of glycogen. *J. Biol. Chem.* 100, 485-491.

[BIO032896C13] Hemre G.-I., Mommsen T. P. and Krogdahl Å. (2002). Carbohydrates in fish nutrition: effects on growth, glucose metabolism and hepatic enzymes. *Aquacult. Nutr.* 8, 175-194. 10.1046/j.1365-2095.2002.00200.x

[BIO032896C14] Hung S. S. O. and Storebakken T. (1994). Carbohydrate utilization by rainbow trout is affected by feeding strategy. *J. Nutr.* 124, 223 10.1093/jn/124.2.223 8308571

[BIO032896C15] Iynedjian P. B. (2009). Molecular physiology of mammalian glucokinase. *Cell. Mol. Life Sci.* 66, 27-42. 10.1007/s00018-008-8322-9 18726182 PMC2780631

[BIO032896C16] Jin J., Medale F., Kamalam B. S., Aguirre P., Veron V. and Panserat S. (2014). Comparison of glucose and lipid metabolic gene expressions between fat and lean lines of rainbow trout after a glucose load. *PLoS ONE* 9, e105548 10.1371/journal.pone.0105548 25141351 PMC4139350

[BIO032896C17] Kamalam B. S., Medale F., Kaushik S., Polakof S., Skiba-Cassy S. and Panserat S. (2012). Regulation of metabolism by dietary carbohydrates in two lines of rainbow trout divergently selected for muscle fat content. *J. Exp. Biol.* 215, 2567-2578. 10.1242/jeb.070581 22786633

[BIO032896C18] Kamalam B. S., Medale F. and Panserat S. (2017). Utilisation of dietary carbohydrates in farmed fishes: new insights on influencing factors, biological limitations and future strategies. *Aquaculture* 467, 3-27. 10.1016/j.aquaculture.2016.02.007

[BIO032896C19] Kaushik S. J., Medale F., Fauconneau B. and Blanc D. (1989). Effect of digestible carbohydrates on protein/energy utilization and on glucose metabolism in rainbow trout (*Salmo gairdneri* R.). *Aquaculture* 79, 63-74. 10.1016/0044-8486(89)90446-8

[BIO032896C20] Kim J. D. and Kaushik S. J. (1992). Contribution of digestible energy from carbohydrates and estimation of protein/energy requirements for growth of rainbow trout (*Oncorhynchus mykiss*). *Aquaculture* 106, 161-169. 10.1016/0044-8486(92)90200-5

[BIO032896C21] Kirchner S., Panserat S., Lim P. L., Kaushik S. and Ferraris R. P. (2008). The role of hepatic, renal and intestinal gluconeogenic enzymes in glucose homeostasis of juvenile rainbow trout. *J. Comp. Physiol. B* 178, 429-438. 10.1007/s00360-007-0235-7 18180932

[BIO032896C22] Kolditz C., Borthaire M., Richard N., Corraze G., Panserat S., Vachot C., Lefèvre F. and Médale F. (2008). Liver and muscle metabolic changes induced by dietary energy content and genetic selection in rainbow trout (*Oncorhynchus mykiss*). *Am. J. Physiol. Regul. Integr. Comp. Physiol.* 294, R1154-R1164. 10.1152/ajpregu.00766.2007 18234747

[BIO032896C23] Lin S.-C. and Hardie D. G. (2017). AMPK: sensing glucose as well as cellular energy status. *Cell Met.* 27, 299-313. 10.1016/j.cmet.2017.10.009 29153408

[BIO032896C24] Liu J., Plagnes-Juan E., Geurden I., Panserat S. and Marandel L. (2017). Exposure to an acute hypoxic stimulus during early life affects the expression of glucose metabolism-related genes at first-feeding in trout. *Sci. Rep.* 7, 363 10.1038/s41598-017-00458-4 28337034 PMC5428409

[BIO032896C25] Marandel L., Seiliez I., Véron V., Skiba-Cassy S. and Panserat S. (2015). New insights into the nutritional regulation of gluconeogenesis in carnivorous rainbow trout (*Oncorhynchus mykiss*): a gene duplication trail. *Physiol. Genomics* 47, 253-263. 10.1152/physiolgenomics.00026.2015 25901068

[BIO032896C26] Marandel L., Dai W., Panserat S. and Skiba-Cassy S. (2016). The five glucose-6-phosphatase paralogous genes are differentially regulated by insulin alone or combined with high level of amino acids and/or glucose in trout hepatocytes. *Mol. Biol. Rep.* 43, 207-211. 10.1007/s11033-016-3962-6 26896939

[BIO032896C27] Mazur C. N., Higgs D. A., Plisetskaya E. and March B. E. (1992). Utilization of dietary starch and glucose tolerance in juvenile Chinook salmon (*Oncorhynchus tshawytscha*) of different strains in seawater. *Fish Physiol. Biochem.* 10, 303-313. 10.1007/BF00004479 24214327

[BIO032896C28] Mommsen T. P. and Plisetkaya E. M. (1991). Fish insulin: history structure and metabolic regulation. *Rev. Aquat. Sci.* 4, 225-229.

[BIO032896C29] Naylor R. L., Hardy R. W., Bureau D. P., Chiu A., Elliott M., Farrell A. P., Forster I., Gatlin D. M., Goldburg R. J., Hua K. et al. (2009). Feeding aquaculture in an era of finite resources. *Proc. Natl. Acad. Sci. USA* 106, 15103-15110. 10.1073/pnas.0905235106 19805247 PMC2741212

[BIO032896C30] Nguyen P., Leray V., Diez M., Serisier S., Le Bloch J., Siliart B. and Dumon H. (2008). Liver lipid metabolism. *J. Anim. Physiol. Anim. Nutr.* 92, 272-283. 10.1111/j.1439-0396.2007.00752.x 18477307

[BIO032896C31] Olsvik P. A., Lie K. K., Jordal A.-E. O., Nilsen T. O. and Hordvik I. (2005). Evaluation of potential reference genes in real-time RT-PCR studies of Atlantic salmon. *BMC Mol. Biol.* 6, 21-29. 10.1186/1471-2199-6-21 16293192 PMC1314898

[BIO032896C32] Panserat S., Médale F., Blin C., Brèque J., Vachot C., Plagnes-Juan E., Gomes E., Krishnamoorthy R. and Kaushik S. (2000a). Hepatic glucokinase is induced by dietary carbohydrates in rainbow trout, gilthead seabream, and common carp. *Am. J. Physiol. Regul. Integr. Comp. Physiol.* 278, R1164-R1170. 10.1152/ajpregu.2000.278.5.R1164 10801283

[BIO032896C33] Panserat S., Médale F., Brèque J., Plagnes-Juan E. and Kaushik S. (2000b). Lack of significant long-term effect of dietary carbohydrates on hepatic glucose-6-phosphatase expression in rainbow trout (*Oncorhynchus mykiss*). *J. Nutr. Biochem.* 11, 22-29. 10.1016/S0955-2863(99)00067-4 15539339

[BIO032896C34] Panserat S., Plagnes-Juan E., Breque J. and Kaushik S. (2001). Hepatic phosphoenolpyruvate carboxykinase gene expression is not repressed by dietary carbohydrates in rainbow trout (*Oncorhynchus mykiss*). *J. Exp. Biol.* 204, 359-365. 11136621 10.1242/jeb.204.2.359

[BIO032896C35] Panserat S., Skiba-Cassy S., Seiliez I., Lansard M., Plagnes-Juan E., Vachot C., Aguirre P., Larroquet L., Chavernac G., Medale F. et al. (2009). Metformin improves postprandial glucose homeostasis in rainbow trout fed dietary carbohydrates: a link with the induction of hepatic lipogenic capacities? *Am. J. Physiol. Regul. Integr. Comp. Physiol.* 297, R707-R715. 10.1152/ajpregu.00120.2009 19553503

[BIO032896C36] Panserat S., Rideau N. and Polakof S. (2014). Nutritional regulation of glucokinase: a cross-species story. *Nutr. Res. Rev.* 27, 21-47. 10.1017/S0954422414000018 24896238

[BIO032896C37] Polakof S., Álvarez R. and Soengas J. L. (2010). Gut glucose metabolism in rainbow trout: implications in glucose homeostasis and glucosensing capacity. *Am. J. Physiol. Regul. Integr. Comp. Physiol.* 299, 19-32. 10.1152/ajpregu.00005.2010 20357022

[BIO032896C38] Polakof S., Mommsen T. P. and Soengas J. L. (2011). Glucosensing and glucose homeostasis: from fish to mammals. *Comp. Biochem. Physiol B* 160, 123-149. 10.1016/j.cbpb.2011.07.006 21871969

[BIO032896C39] Polakof S., Panserat S., Soengas J. L. and Moon T. W. (2012). Glucose metabolism in fish: a review. *Comp. Biochem. Physiol B* 182, 1015-1045. 10.1007/s00360-012-0658-7 22476584

[BIO032896C40] Quillet E., Le Guillou S., Aubin J. and Fauconneau B. (2005). Two-way selection for muscle lipid content in pan-size rainbow trout (*Oncorhynchus mykiss*). *Aquaculture* 245, 49-61. 10.1016/j.aquaculture.2004.12.014

[BIO032896C41] Quillet E., Dorson M., Le Guillou S., Benmansour A. and Boudinot P. (2007). Wide range of susceptibility to rhabdoviruses in homozygous clones of rainbow trout. *Fish Shellfish Immunol.* 22, 510-519. 10.1016/j.fsi.2006.07.002 17085058

[BIO032896C42] Sadoul B., Friggens N. C., Valotaire C., Labbé L., Colson V., Prunet P. and Leguen I. (2017). Physiological and behavioral flexibility to an acute CO_2_ challenge, within and between genotypes in rainbow trout. *Comp. Biochem. Physiol. A* 209, 25-33. 10.1016/j.cbpa.2017.04.002 28396262

[BIO032896C43] Seiliez I., Panserat S., Lansard M., Polakof S., Plagnes-Juan E., Surget A., Dias K., Larquier M., Kaushik S. and Skiba-Cassy S. (2011). Dietary carbohydrate-to-protein ratio affects TOR signaling and metabolism-related gene expression in the liver and muscle of rainbow trout after a single meal. *Am. J. Physiol. Regul. Integr. Comp. Physiol.* 300, R733-R743. 10.1152/ajpregu.00579.2010 21209382

[BIO032896C44] Shiau S. Y. and Lin Y. H. (2001). Carbohydrate utilization and its protein-sparing effect in diets for grouper (*Epinephelus malabaricus*). *Anim. Sci.* 73, 299-304. 10.1017/S1357729800058276

[BIO032896C45] Shiau S.-Y. and Peng C.-Y. (1993). Protein-sparing effect by carbohydrates in diets for tilapia, *Oreochromis niloticus*×*O. aureus*. *Aquaculture* 117, 327-334. 10.1016/0044-8486(93)90329-W

[BIO032896C46] Skiba-Cassy S., Lansard M., Panserat S. and Médale F. (2009). Rainbow trout genetically selected for greater muscle fat content display increased activation of liver TOR signaling and lipogenic gene expression. *Am. J. Physiol. Regul. Integr. Comp. Physiol.* 297, R1421-R1429. 10.1152/ajpregu.00312.2009 19710390

[BIO032896C47] Skiba-Cassy S., Panserat S., Larquier M., Dias K., Surget A., Plagnes-Juan E., Kaushik S. and Seiliez I. (2013). Apparent low ability of liver and muscle to adapt to variation of dietary carbohydrate: protein ratio in rainbow trout (*Oncorhynchus mykiss*). *Br. J. Nutr.* 109, 1359-1372. 10.1017/S0007114512003352 22951215

[BIO032896C48] Sundby A., Hemre G.-I., Borrebaek B., Christophersen B. and Blom A. K. (1991). Insulin and glucagon family peptides in relation to activities of hepatic hexokinase and other enzymes in fed and starved Atlantic salmon (*Salmo salar*) and cod (*Gadus morhua*). *Comp. Biochem. Physiol B* 100, 467-470. 10.1016/0305-0491(91)90205-R 1814675

[BIO032896C49] The National Research Council (NRC). (2011). *Nutrient Requirements of Fish and Shrimp*. The National Academies Press: Washington DC, USA.

[BIO032896C50] Towle H. C., Kaytor E. N. and Shih H.-M. (1997). Regulation of the expression of lipogenic enzyme genes by carbohydrate. *Annu. Rev. Nutr.* 17, 405-433. 10.1146/annurev.nutr.17.1.405 9240934

[BIO032896C51] van de Werve G., Lange A., Newgard C., Méchin M.-C., Li Y. and Berteloot A. (2000). New lessons in the regulation of glucose metabolism taught by the glucose 6-phosphatase system. *Eur. J. Biochem.* 267, 1533-1549. 10.1046/j.1432-1327.2000.01160.x 10712583

[BIO032896C52] Wilson R. P. (1994). Utilization of dietary carbohydrate by fish. *Aquaculture* 124, 67-80. 10.1016/0044-8486(94)90363-8

[BIO032896C53] Yamamoto T., Konishi K., Shima T., Furuita H., Suzuki N. and Tabata M. (2001). Influence of dietary fat and carbohydrate levels on growth and body composition of rainbow trout *Oncorhynchus mykiss* under self-feeding conditions. *Fish Sci.* 67, 221-227. 10.1046/j.1444-2906.2001.00243.x

[BIO032896C54] Zhang B. B., Zhou G. and Li C. (2009). AMPK: an emerging drug target for diabetes and the metabolic syndrome. *Cell Metab.* 9, 407-416. 10.1016/j.cmet.2009.03.012 19416711

